# Systematic review of gastric cancer-associated genetic variants, gene-based meta-analysis, and gene-level functional analysis to identify candidate genes for drug development

**DOI:** 10.3389/fgene.2022.928783

**Published:** 2022-08-16

**Authors:** Sangjun Lee, Han-Kwang Yang, Hyuk-Joon Lee, Do Joong Park, Seong-Ho Kong, Sue K. Park

**Affiliations:** ^1^ Department of Preventive Medicine, Seoul National University College of Medicine, Seoul, South Korea; ^2^ Cancer Research Institute, Seoul National University College of Medicine, Seoul, South Korea; ^3^ Department of Biomedical Sciences, Seoul National University Graduate School, Seoul, South Korea; ^4^ Department of Surgery and Cancer Research Institute, Seoul National University College of Medicine, Seoul, South Korea; ^5^ Integrated Major in Innovative Medical Science, Seoul National University College of Medicine, Seoul, South Korea

**Keywords:** stomach neoplasms, gastric cancer, genome-wide association study, gene-based analysis, functional annotations

## Abstract

**Objective:** Despite being a powerful tool to identify novel variants, genome-wide association studies (GWAS) are not sufficient to explain the biological function of variants. In this study, we aimed to elucidate at the gene level the biological mechanisms involved in gastric cancer (GC) development and to identify candidate drug target genes.

**Materials and methods:** We conducted a systematic review for GWAS on GC following the PRISMA guidelines. Single nucleotide polymorphism (SNP)-level meta-analysis and gene-based analysis (GBA) were performed to identify SNPs and genes significantly associated with GC. Expression quantitative trait loci (eQTL), disease network, pathway enrichment, gene ontology, gene-drug, and chemical interaction analyses were conducted to elucidate the function of the genes identified by GBA.

**Results:** A review of GWAS on GC identified 226 SNPs located in 91 genes. In the comprehensive GBA, 44 genes associated with GC were identified, among which 12 genes (*THBS3, GBAP1, KRTCAP2, TRIM46, HCN3, MUC1, DAP3, EFNA1, MTX1, PRKAA1, PSCA*, and *ABO*) were eQTL. Using disease network and pathway analyses, we identified that *PRKAA*, *THBS3*, and *EFNA1* were significantly associated with the PI3K-Alt-mTOR-signaling pathway, which is involved in various oncogenic processes, and that *MUC1* acts as a regulator in both the PI3K-Alt-mTOR and P53 signaling pathways. Furthermore, *RPKAA1* had the highest number of interactions with drugs and chemicals.

**Conclusion:** Our study suggests that *PRKAA1*, a gene in the PI3K-Alt-mTOR-signaling pathway, could be a potential target gene for drug development associated with GC in the future.

**Systematic Review Registration**: website, identifier registration number.

## 1 Introduction

Gastric cancer (GC) was the cancer with the fifth-highest worldwide incidence in 2020, with 1,089,103 new cases (Sung et al., 2021). The incidence of GC is highly variable depending on the region and culture, with the highest incidence rates in Eastern Asia, Europe, and South America (Sung et al., 2021). In Eastern Asia, the average incidence of GC is 32.5 per 100,000 among males and 13.2 among females. On the contrary, in North America, the overall incidence among males and females is 5.4 and 3.1 per 100,000, respectively. The lowest incidence is in regions of Middle Africa, where only 4.6 per 100,000 males and 3.8 per 100,000 females are diagnosed annually. (Sung et al., 2021).

The sequencing and bioinformatic advances in the past decade have permitted genome-wide association studies (GWAS) to become an innovative tool for identifying new single nucleotide polymorphisms (SNPs) or genes for cancer susceptibility (Wang et al., 2005). GWAS explore the associations between a large number of SNPs and traits such as major diseases, thereby investigating the entire genome with an unbiased approach (Manolio, 2010). Previous GWAS and meta-analyses have identified several genetic variants that are associated with GC susceptibility (Mocellin et al., 2015; Jin et al., 2020; Yan et al., 2020). However, no systematic reviews have evaluated the genetic factors associated with GC using gene-based meta-analyses or gene-network analyses.

Despite GWAS being powerful tools for the identification of novel variants associated with a certain trait, they may not capture the entire signal due to a lack of power, and their results may be biased due to population stratification or locus heterogeneity (Luo et al., 2010). In addition, as the identified variants may be non-pathogenic variants in linkage disequilibrium (LD) with the actual causal variants, follow-up studies are necessary to confirm the functional effects of the identified signal (Stadler et al., 2010).

Gene-based analysis (GBA) has recently been suggested as an approach to overcome the limitations of GWAS. GBA can detect regions that display allelic heterogeneity and identify modest genetic effects by improving statistical power by combining single variants obtained from individual GWAS (Liu et al., 2010; Huang et al., 2011). Another approach to overcome the limitations of GWAS is expression quantitative trait loci (eQTL) analysis. This method permits the functional interpretation of GWAS markers by linking them to changes in gene expression (Nica and Dermitzakis, 2013). Furthermore, pathway and Gene Ontology (GO) enrichment analyses of the identified variants can inform about the biological function of the identified variants at the gene level (Gene Ontology Consortium, 2015; Slenter et al., 2018). Finally, as genes associated with a specific disease can be pleiotropic, meaning that they can be associated with other diseases or phenotypes (Solovieff et al., 2013). Disease interaction analysis has also been conducted to identify shared pathological pathways (Piñero et al., 2020). Using a combination of these approaches, studying genetic variants and their functions in disease can ultimately be used to identify novel drug targets or biomarkers (Wheeler et al., 2013).

The purpose of this study was to identify potential genes for drug development associated with GC based on a comprehensive understanding of the biological mechanisms of GC-associated genes by systematically reviewing published GWAS for GC and performing gene-level functional analyses, including drug/chemical interactions, through GBA.

## 2 Materials and methods

### 2.1 Literature search and selection criteria

Our study conducted a systematic review according to the Preferred Reporting Items for Systematic Reviews and Meta-Analysis (PRISMA) guidelines (Supplementary Table S1). (Page et al., 2021) The inclusion criteria based on the Population, Intervention, Comparison, Outcome, Study design (PICOS) model were as follows (Richardson et al., 1995): 1) Population: Human patients with gastric cancer; 2) Intervention (Exposure): Genetic variants (SNPs); 3) Comparison: Control group with unaffected risk alleles; 4) Outcome: Genotyping profiles of patients with gastric cancer; 5) Study design: case-control, GWAS. We excluded all studies that were not published in English and did not perform the GWAS analysis. In addition, studies in which the GWAS analysis was repeated with the same population were also excluded. An overall identification of eligible studies on the literature search was presented in the PRISMA2020 flow diagram (Supplementary Figure S1) (Haddaway et al., 2022).

To retrieve potentially eligible studies from PubMed and Embase, combinations of search queries were used (Supplementary Table S2). A PubMed search was conducted using “RISmed” R package, whereas Embase search was conducted in online search (https://www.embase.com/search/quick, assessed on 20 March 2021) (Kovalchik, 2014). In addition, a detailed search of several publicly available GWAS databases (registers) was conducted: the Human Genome Epidemiology (HuGE) Navigator (https://phgkb.cdc.gov/PHGKB/hNHome.action), the Genome-Wide Repository of Associations between SNPs and Phenotypes (GRASP) (https://grasp.nhlbi.nih.gov/Search.aspx), the National Human Genome Research Institute (NHGRI GWAS Catalog) (https://www.ebi.ac.uk/gwas/), and the GWAS Central (https://www.gwascentral.org/). All GWAS databases (registers) were assessed on 20 March 2021. We only included articles for which the full text was published on or before 31 December 2020. The identification of all studies was performed by two independent researchers. The two independent researchers independently extracted data on the first author’s name, publication year, study design, location of the study, ethnicity of the participants, number of cases/controls, SNPs investigated, chromosome, candidate genes, genotyping platform used, cancer type/location, measure of association with corresponding to 95% confidence interval (CI), and *p*-value obtained from the combined sample sets.

Non-Randomised Studies of Interventions (ROBINS-I) tool was used to assess the risk of bias (RoB) in non-randomized studies (Sterne et al., 2016). The instrument was developed to assess the internal validity of non-randomized trials by assessing the RoB within the seven domains: 1) Confounding bias, 2) Bias in the selection of study participants, 3) Bias in classification of intervention, 4) Bias due deviation from intended intervention, 5) Bias due to missing data, 6) Bias in measurement of outcome, and 7) Bias in the selection of reported results. The domain conclusion classified the overall body of evidence into “low”, “moderate”, “serious”, and “critical” categories. The results were also visualized by “robvis” Shiny (McGuinness and Higgins, 2021).

When the opinions of the two independent researchers differed, the four co-authors who are gastrointestinal surgery clinicians were consulted to resolve the dispute. We followed the principles proposed by the Human Genome Epidemiology Network (HuGeNet) for a systematic review of molecular association studies (Little et al., 2006).

### 2.2 Meta-analysis

A meta-analysis was performed to synthesize a total of 522 SNPs associated with GC that were included in 12 eligible studies as follows (Gurevitch et al., 2018):

1) The original values of the fixed-effect model were obtained when the reported SNPs in the individual study were validated several times.
2) The values of the fixed-effect model were estimated when external replication was possible because each SNP was reported only once in each different study.
3) The original odds ratios (ORs) (95% CI) or P-values for the single SNPs reported in the eligible studies were also obtained even though those of SNPs were excluded from the gene-based analysis.
4) When the same SNPs were reported from GC and its sub-types, the ORs (95% CI) or P-values reported for GC were used.
5) When the OR (95% CI) or P-value for the same SNP was estimated from multiple subtypes, the one with a lower P-value was used for the meta-analysis.
6) The OR (95% CI) and P-value were calculated based on the random-effect model. However, the values of the random-effect model could not be estimated when the OR was reported only once or when only the OR *p*-value was presented for each SNP.

ORs were calculated for each study and polymorphism assuming an additive genetic model. Heterogeneity in the meta-analysis was evaluated using I^2^ statistics (Higgins and Thompson, 2002). All statistical analyses were performed using the R software (version 4.1.0).

### 2.3 Gene-based analysis: Burden test

Gene-level association tests in the random-effects model were performed after weighting by minor-allele frequencies (MAFs) (Morgenthaler and Thilly, 2007). We combined information across several variants in a target region and then performed a burden test based on a single/meta-analysis for each SNP with LD structure based on the 1,000 Genome reference panel (Phase 3, East Asian). The burden test results were converted to gene-level estimates of effect sizes (betas) and their standard errors (Svishcheva et al., 2015). When multiple SNPs were in high LD (*R* ^2^ > 0.9) in the same gene region, a burden test was performed with SNPs remaining after LD clumping. A Bonferroni correction for multiple testing was applied to account for the total number of genes tested (approximately 20,000 genes). Significant gene-level associations in the burden test were those with a *p*-value < 2.5 × 10^–6^ after correcting for multiple testing.

### 2.4 Functional annotation analysis

#### 2.4.1 eQTL analysis

Overlapping eQTL analysis was performed to identify SNPs affecting a regulatory element controlling gene expression (Nica and Dermitzakis, 2013). The eQTL were identified based on the eQTLGen consortium, which is a large-scale multi-study effort to identify the downstream effects of trait-related variants via their effects on gene expression in whole blood (Võsa et al., 2018). eQTL analysis was also conducted based on the Genotype-Tissue Expression (GTEx) project, which aims to study tissue-specific gene expression and regulation (Carithers and Moore, 2015). We used individual-level data in stomach tissue from GTEx (v8) to construct the co-expression matrix and further validate the gene sets reported by eQTLGen.

We performed eQTL analysis based on SNP statistics (*p*-value) from the meta-analysis and burden test using Functional Mapping and Annotation of Genome-Wide Association Studies (FUMA) online software (https://fuma.ctglab.nl/) (Watanabe et al., 2017). Significant eQTL with a False Discovery Rate (FDR) ≤ 0.05 were selected for further analysis. Gene annotation was performed based on the Genome Reference Consortium Human Genome Build 37 or hg19 reference assembly.

#### 2.4.2 Disease network analysis

The disease network analysis was used to identify candidate genes for GC from a burden test using DisGeNET (Piñero et al., 2020). DisGeNET is a discovery platform that contains one of the largest publicly available collections of genes and variants associated with humans. An FDR-corrected *p*-value of <0.05 was used to identify significant disease networks.

#### 2.4.3 Pathway analysis

To identify pathways associated with GC, we used statistical results from the WikiPathway Human Collection (http://wikipathways.org) (Slenter et al., 2018) and the Network Data Exchange (NDEx) (https://www.ndexbio.org/) (Pratt et al., 2015). The WikiPathway is a collaborative open database that includes knowledge of curated biological pathways. In addition, the NDEx database provides access to not only pathways but also diverse types of network models, offering digital object identifier (DOI) minting for citation. Pathways with an FDR <0.05, including at least one altered gene, were considered significant.

#### 2.4.4 GO analysis

We performed GO analysis to annotate genes to known functional information sources (Gene Ontology Consortium, 2015), including biological process (BP), cellular component (CC), and molecular function (MF) using the “clusterProfilter” R package (Yu et al., 2012). We submitted genes significantly estimated from the burden test and were considered significant for GO results with an FDR <0.05.

#### 2.4.5 Gene-drug interaction analysis

We studied gene-drug interactions using the DrugBank database (https://go.drugbank.com/) and the DGIdb database (http://www.dgidb.org/) (Wishart et al., 2018; Freshour et al., 2021). DrugBank is a drug-centric online database that provides detailed information about over 500,000 drugs and their target genes. DGIdb comprises drug-gene interaction information of more than 40,000 genes and 10,000 drugs from 15 different resources and allows filtering at different levels. Only gene-drug interactions in which the drug was found in two or more references or databases were selected.

#### 2.4.6 Gene-chemical interaction analysis

The Comparative Toxicogenomics Database (CTD) (http://ctdbase.org/) was employed to construct a gene-chemical interaction network (Davis et al., 2021). The CTD includes toxicological information for over 16,000 chemicals and 50,000 genes. Only gene-chemical interactions with two or more references were selected.

#### 2.4.7 Protein-protein interaction analysis

The Search Tool for the Retrieval of Interacting Genes (STRING; http://string.embl.de/) is a biological database designed to construct a PPI network by analyzing the functional interactions between proteins (Szklarczyk et al., 2021). Using STRING, PPIs were constructed with a confidence score ≥0.99 (Asadzadeh-Aghdaee et al., 2016). Subsequently, the PPI network was visualized using the Cytoscape software (version 3.8.2) (Shannon et al., 2003) via Rcy3 (Gustavsen et al., 2019).

## 3 Results

### 3.1 Flow of study selection

We identified 3,251 and 90 eligible studies through PubMed and Embase, respectively (Supplementary Figure S1). In addition, 906, 123, 46, and 14 eligible studies were also identified through the HUGE Navigator, GRASP, GWAS Catalog, and GWAS Central, respectively (Supplementary Figure S1). Subsequently, 230 duplicated studies and two studies that were written in other languages were removed. After title and abstract screening, 348 full text articles were assessed for further eligibility. A total of 333 studies were excluded for not conducting GWAS, and three full text articles were excluded for repeating the analyses in the same population. The remaining 12 GWAS for GC, including duplicated 522 SNPs, were included in the meta-analysis (Supplementary Figures S1, S2) (Sakamoto et al., 2008; Abnet et al., 2010; Shi et al., 2011; Jin et al., 2012; Tanikawa et al., 2012; Helgason et al., 2015; Hu et al., 2016; Wang et al., 2017; Tanikawa et al., 2018; Park et al., 2019; Du et al., 2020; Rashkin et al., 2020). Among the selected studies, ten were conducted in Asia (China, Japan, Korea, and Singapore), and two were performed in Europe and North America. The studies were published between 2008 and 2020. The present study was approved by the respective institutional ethics review committee, and informed consent was obtained from all participants.

### 3.2 Study characteristics and risk of bias within the studies

Of the total of 12 studies, ten of which focused on Asians (Korea, China, Japan, Singapore) (Sakamoto et al., 2008; Abnet et al., 2010; Shi et al., 2011; Jin et al., 2012; Tanikawa et al., 2012; Hu et al., 2016; Wang et al., 2017; Tanikawa et al., 2018; Park et al., 2019; Du et al., 2020), one in Europe (Helgason et al., 2015), and the other in the United States/United Kingdom (Rashkin et al., 2020). 12 studies in all were adjusted for age and sex or additional covariates such as principal components. Three studies presented the results of the diffuse and intestinal subtypes (Sakamoto et al., 2008; Tanikawa et al., 2012; Tanikawa et al., 2018), while five studies revealed the results of cardia or non-cardia subtypes (Abnet et al., 2010; Shi et al., 2011; Jin et al., 2012; Hu et al., 2016; Wang et al., 2017). Furthermore, two studies presented findings associated with adenocarcinoma (Abnet et al., 2010; Helgason et al., 2015). Other studies without subtype analysis have reported GC results.

The assessment of RoB for observational studies was shown in Supplementary Table S3 and Supplementary Figure S3. Based on the ROBINS-I tool, 4 studies were identified as “low risk”, 6 studies were assessed as “moderate risk” studies, while 2 studies were considered as “Serious risk”. The *p*-value of each SNP reported prior to data synthesis in one of the two severe risk studies was not genome-wide significant (5 × 10^–8^) (Sakamoto et al., 2008), and some of the SNP annotations and effect sizes were not presented in another study (Jin et al., 2012). It is believed that there may be limitations because this GWAS study is in its early days. GWAS generally adjusts for age and sex, but if there is heterogeneity in the population, the principal component is additionally adjusted (McCaw et al., 2022). However, in our eligible studies, validation analysis was also performed with the same ethnicity, so there seems to be little bias due to the confounder. According to our assessment of the certainty of the evidence, the body of evidence supporting an association between SNPs and elevated risk of GC had “moderate degree of evidence”.

### 3.3 Major genes associated with GC: Meta-and gene-based analyses

**FIGURE 1:**
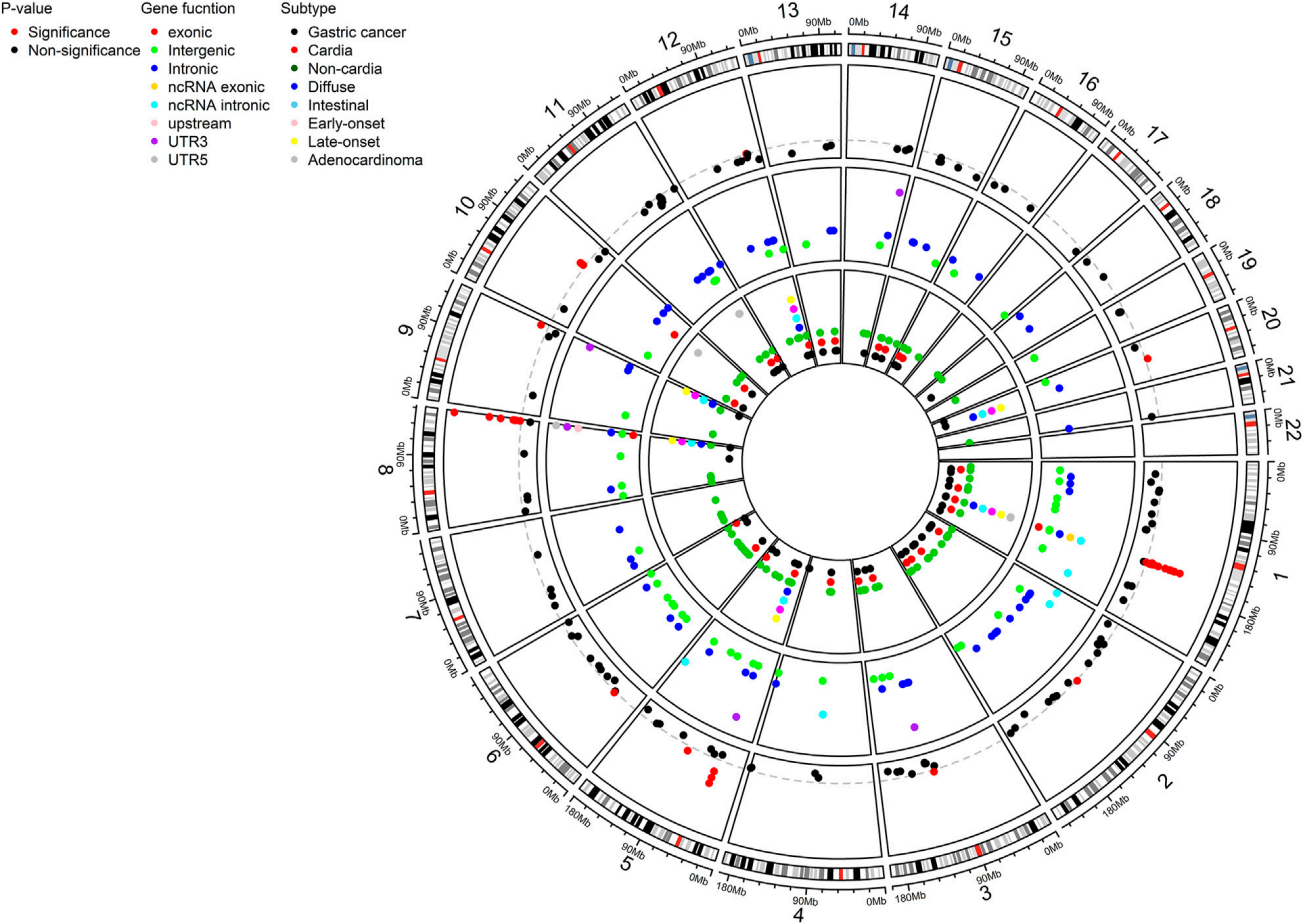
Circos plot showing a total of 226 distinct SNPs for gastric cancer susceptibility as a result of systematic review and meta-analysis. SNPs were shown by P-value (significant threshold: 5 × 10^-8^), genetic function, and subtypes. SNP, single nucleotide polymorphism; UTR, untranslated region.

A total of 552 SNPs were identified from the eligible studies based on literature search (Supplementary Table S4). 522 SNPs were located in upstream (n = 5), downstream (n = 9), intronic (n = 207), exonic (n = 28), noncoding RNA (ncRNA) intronic (n = 10), ncRNA exonic (n = 8), 5′-UTR (n = 15), 3′-UTR (n = 38), and intergenic (n = 202) regions (Figure 1; Supplementary Table S4). Some of the SNPs were associated specifically with histological subtypes (intestinal; n = 12, diffuse; n = 24), site (cardia; n = 36, non-cardia; n = 98), onset age (early; n = 6, late; n = 6), and pathological subtype (adenocarcinoma; n = 17) (Figure 1; Supplementary Table S4). Out of 522 SNPs, 296 SNPs were reported in the multiple studies or overlapped results from subtypes were excluded (Supplementary Table S5). Therefore, a total of 226 SNPs remained in the meta-analysis based on both the fixed- and random-effect models (Supplementary Table S6). In many cases, 25%–49%, 50%–74%, and over 75% of I^2^ suggest low, intermediate, and high heterogeneity, respectively (Higgins et al., 2003). Among 226 SNPs, 41 SNPs had no heterogeneity. On the other hand, 24, 71, and 53 SNPs had low, intermediate, and high heterogeneity, respectively. The heterogeneity of 37 SNPs was not evaluated due to without validation (Supplementary Table S6).

Since a gene’s effect size is estimated based on the effect size of several SNPs located in the gene, 59 SNPs located in the intergenic position were excluded from the remaining 226 SNPs to perform the gene-based analysis. Therefore, 167 SNPs located in 91 genes were retained as candidates for gene-based analysis (Supplementary Figure S2). Among the 91 genes, 44 genes were included in the burden test after excluding genes that were specifically associated with a subgroup of GC or in a non-Asian population, genes whose effect size was estimated from a single distinct SNP, and genes not identified as entrez id (Supplementary Figure S2). Of the 44 genes, effect sizes for six genes and 38 genes were estimated by the burden test and the meta-analysis, respectively (Table 1). After correcting for multiple testing, 25 genes had significant gene-level associations with GC (*p*-value < 2.5 × 10^–6^).

**TABLE 1:** The results of gene-based analysis for gastric cancer.

Gene	Chr	OR (95% CI)	*p*-value	Method
*KRTCAP2*	1q22	0.65 (0.60–0.71)	8.44E-24	Burden Test
*MUC1*	1q22	0.76 (0.71–0.81)	1.99E-14	Meta
*MTX1*	1q22	0.71 (0.63–0.80)	1.70E-08	Burden Test
*GBAP1*	1q22	0.48 (0.42–0.55)	1.03E-27	Burden Test
*EFNA1*	1q22	0.82 (0.77–0.87)	1.46E-10	Meta
*TRIM46*	1q22	0.71 (0.66–0.76)	1.65E-21	Burden Test
*THBS3*	1q22	0.51 (0.45–0.57)	6.39E-29	Burden Test
*HCN3*	1q22	0.83 (0.80–0.87)	2.65E-17	Meta
*DAP3P1*	1q22	0.64 (0.56–0.74)	1.56E-10	Meta
*MST O 1*	1q22	0.67 (0.60–0.75)	1.60E-12	Meta
*DAP3*	1q22	0.65 (0.58–0.73)	4.23E-13	Meta
*GON4L*	1q22	0.63 (0.55–0.72)	4.47E-11	Meta
*RPL7P10*	1p31.1	1.25 (1.10–1.42)	5.76E-04	Meta
*SERINC2*	1p35.2	1.01 (0.96–1.06)	8.05E-01	Meta
*RMDN2*	2p22.2	0.67 (0.58–0.78)	3.24E-07	Meta
*LYPD6*	2q23.2	0.84 (0.77–0.91)	4.27E-05	Meta
*BAZ2B*	2q24.2	1.29 (1.14–1.45)	2.59E-05	Meta
*BZW1*	2q33.1	0.97 (0.93–1.01)	1.56E-01	Meta
*UMPS*	3q21.2	1.03 (0.99–1.08)	2.00E-01	Meta
*ITGB5*	3q21.2	0.95 (0.91–0.99)	2.92E-02	Meta
*TRIML1*	4q35.2	0.73 (0.64–0.84)	1.68E-05	Meta
*RAB3C*	5q11.2	1.07 (1.03–1.13)	2.46E-03	Meta
*PRKAA1*	5p13.1	0.80 (0.77–0.83)	4.83E-26	Meta
*GPX3*	5q33.1	0.92 (0.89–0.95)	1.49E-06	Meta
*LINC01411*	5q35.2	1.05 (1.01–1.10)	2.65E-02	Meta
*UNC5CL*	6p21.1	1.14 (1.09–1.20)	7.22E-08	Meta
*SAMD5*	6q24.3	0.81 (0.75–0.88)	5.62E-07	Meta
*PSCA*	8q24.3	0.75 (0.72–0.78)	8.20E-56	Meta
*ABO*	9q34.11	1.15 (1.11–1.19)	2.64E-13	Meta
*PLCE1*	10q23.33	3.60 (2.46–5.26)	3.51E-11	Burden Test
*LOC101928477*	11q22.2	0.95 (0.84–1.08)	4.29E-01	Meta
*DYNC2H1*	11q22.3	0.95 (0.91–1.00)	3.30E-02	Meta
*OPCML*	11q25	1.19 (1.11–1.28)	1.60E-06	Meta
*CCDC63*	12q24.11	0.92 (0.89–0.96)	1.80E-05	Meta
*CUX2*	12q24.12	0.91 (0.88–0.94)	3.20E-08	Meta
*DTX1*	12q24.13	1.15 (1.09–1.21)	1.20E-07	Meta
*GPC5*	13q31.3	1.07 (1.02–1.11)	2.31E-03	Meta
*UBAC2*	13q32.3	1.07 (1.03–1.11)	2.71E-04	Meta
*FMN1*	15q13.3	1.11 (1.05–1.17)	4.53E-04	Meta
*TRPM1*	15q13.3	0.61 (0.50–0.74)	5.87E-07	Meta
*RORA*	15q22.2	1.09 (1.04–1.14)	1.83E-04	Meta
*SNX29 *	16p13.13	0.92 (0.88–0.96)	1.83E-04	Meta
*HA O 1*	20p12.3	0.92 (0.87–0.96)	7.20E-04	Meta
*DEFB121*	20q11.21	1.11 (1.07–1.15)	8.11E-10	Meta

OR, odds ratio; CI, confidence interval; BT, burden test.

### 3.4 Functional annotation analysis

eQTL analysis was performed based on 226 SNPs with statistics (*p*-value) after the meta-analysis. The eQTL analysis results were represented in SNP-gene pairs since the SNPs have a role in gene expression regulation. Furthermore, since one SNP can affect the expression level of multiple genes, the results of eQTL analysis were calculated in pairs.

Forty-seven SNPs out of the 226 SNPs in the meta-analysis were identified to regulate the expression of 12 genes (*THBS3, GBAP1, KRTCAP2, TRIM46, HCN3, MUC1, DAP3, EFNA1, MTX1, PRKAA1, PSCA*, and *ABO*) out of the 25 genes significantly estimated from the burden tests, resulting in a total of 175 SNP-gene pairs (Supplementary Table S7). In *PRKAA1*, *PSCA*, and *ABO*, the SNPs located in the corresponding gene regulated the expression of their respective (Supplementary Figure S4). Three pairs (three SNPs-one gene) for *PRKAA1*, 26 pairs (26 SNPs-one gene) for *PSCA*, and one pair (one SNP-one gene) for *ABO* were estimated (Supplementary Figure S4). However, the expression level of nine genes (*THBS3*, *GBAP1*, *KRTCAP2*, *TRIM46*, *HCN3*, *MUC1*, *DAP3*, *EFNA1*, and *MTX1*) on chromosome 1 were regulated by 17 SNPs located nearby (Supplementary Figure S4; Supplementary Table S7). Among the 17 SNPs, 13 (rs1057941, rs12752585, rs2049805, rs28445596, rs2974929, rs2990220, rs4276914, rs4971059, rs4971085, rs4971088, rs4971100, rs4971101, and rs7556304) regulated the expression of 9 genes, yielding a total of 117 pairs. Three SNPs (rs2066981, rs3814316, and rs4971093) regulated the expression of eight genes, not including *EFNA1*, yielding 24 pairs. Finally, rs4971066 regulated the expression level of only four genes (*GBAP1*, *THBS3*, MTX1, and *MUC1*), establishing four pairs. In total, eQTL analysis yielded 175 SNP-gene pairs between 47 SNPs and 12 genes.

Cis-eQTL identification based on the eQTLGen database yielded a total of 120 significant (FDR ≤0.05) pairs between 21 SNPs and 11 genes (*THBS3, GBAP1, KRTCAP2, TRIM46, HCN3, MUC1, DAP3, EFNA1, MTX1, PRKAA1*, and *ABO*). In contrast, no trans-eQTL were found. eQTL refers to genetic variants involved in regulating gene expression (Võsa et al., 2021). eQTL is divided into cis-eQTL and trans-eQTL. SNPs regulating gene expression located near a gene (<1 megabase; Mb) with local effects are called cis-QTL, whereas SNPs located distally (>5 Mb) or on a different chromosome of a gene with remote effects are called trans-eQTLs (Westra and Franke, 2014). Because cis-eQTLs generally have large effect sizes (Huang da et al., 2009), even moderate sample size enables the detection of cis-eQTLs of thousands of genes (Westra et al., 2013). In addition, cis-eQTLs have a direct effect on gene expression due to their proximity to the transcription start site (TSS) (Stranger et al., 2012). On the other hand, since the effect size of trans-eQTLs is generally small, a larger sample size is required (Grundberg et al., 2012). Moreover, it is hard to identify validated reports to estimate the effect size for tans-eQTLs due to estimation difficulty (Westra et al., 2013). Nevertheless, since a trans-eQTL can affect multiple genes with small effect size and can have a wide range of effects in biological networks, it can be highly associated with a cross-phenotype (Brynedal et al., 2014; Westra and Franke, 2014).

Based on the GTEx-stomach database, 55 pairs between 42 SNPs and three genes (*THBS3, GBAP1*, and *PSCA*) were significant (FDR ≤0.05) (Supplementary Table S7). The results for *THBS3* and *GBAP1* were validated on both the eQTLGen and GTEx-stomach databases.

**FIGURE 2:**
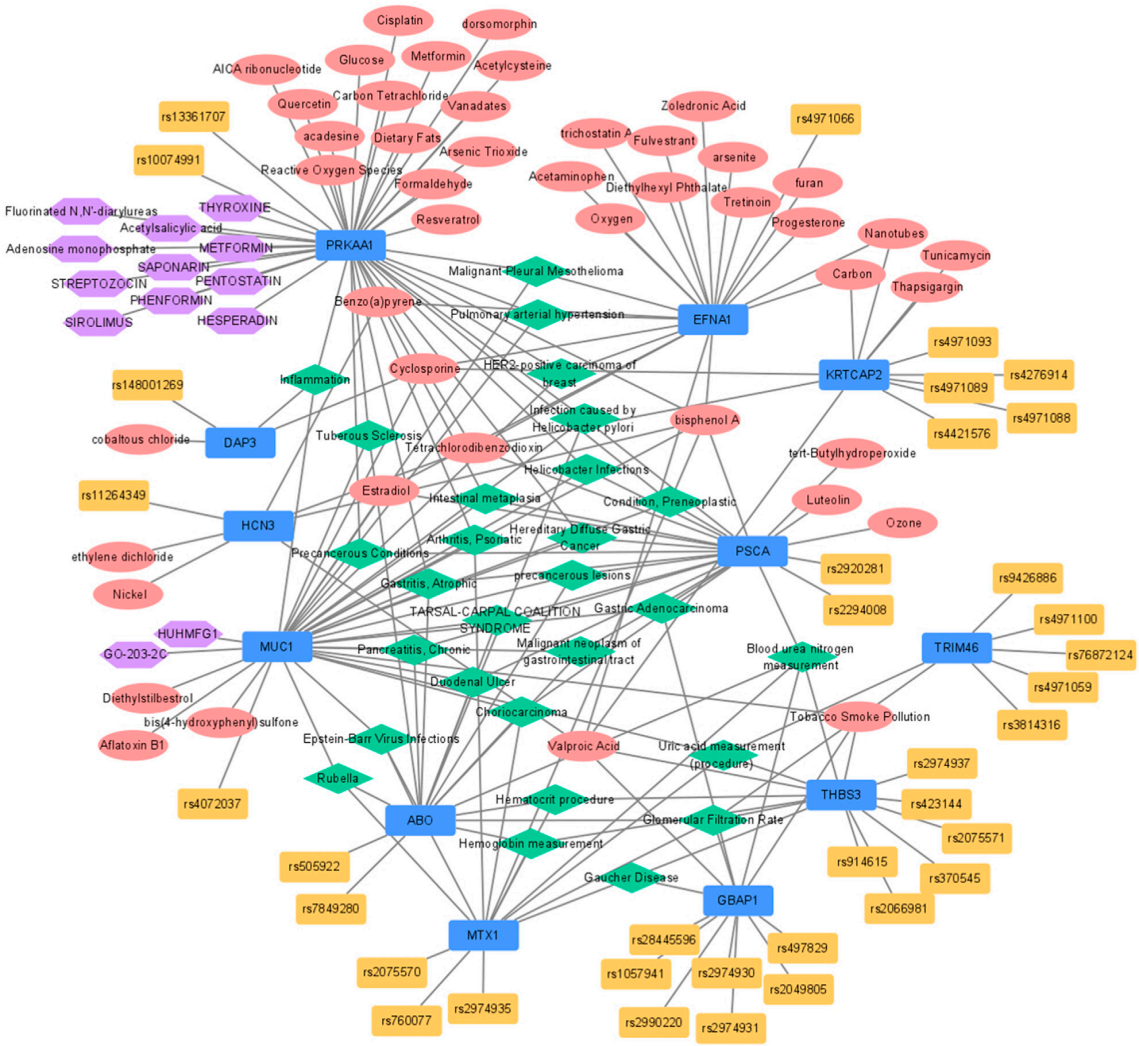
Functional network analysis for expression-regulated genes from eQTL identified by GBA. Blue, red, yellow, green, and purple nodes stood for genes, chemicals, SNPs included in GBA, disease-networks, and drugs, respectively. eQTL, expression quantitative trait loci; GBA, gene-based analysis; SNP, single nucleotide polymorphism.

Of the 12 eQTL genes, 10 were associated with a total of 28 diseases according to the disease network analysis (Figure 2; Supplementary Figure S5). Among these diseases, 11 were associated with GC (*Helicobacter pylori* infections, infection caused by *Helicobacter pylori*, atrophic gastritis, duodenal ulcer, preneoplastic conditions, intestinal metaplasia, precancerous lesions, hereditary diffuse gastric cancer, malignant neoplasm of gastrointestinal tract, gastric adenocarcinoma, and precancerous conditions). In addition, three biomarkers of chronic kidney disease were identified (blood urea nitrogen, glomerular filtration rate, and uric acid). Five diseases associated with uric acid or inflammation (Gaucher disease, tarsal-carpal coalition syndrome, tuberous sclerosis, psoriatic arthritis, and inflammation) and two viral diseases were selected (Rubella and Epstein-Barr virus infection), and hemoglobin and hematocrit were also found in the disease network. Additionally, five other diseases associated with eQTL genes were identified (Supplementary Figure S5).

A total of 18 pathways were significantly associated with 12 eQTL genes in Wikipathways (Figure 2; Supplementary Figure S5). Among these pathways, the PI3K-Alt-mTOR-signaling pathway containing three eQTL genes (*THBS3, EFNA1*, and *PRKAA1*) was the most significant (FDR = 5.99 × 10^–4^). *PRKAA1* was associated with 13 pathways and was the gene that showed the strongest association among the 12 eQTL genes (FDR <0.05). According to the NDEx database, *MUC1* was identified as a regulator of the PI3K-Alt-mTOR and p53 signaling pathways. Moreover, RAS drives the PI3K-Alt-mTOR-signaling pathway via PRKAA1, the adenosine monophosphate-activated kinase (AMPK) via the ERK pathway, and TP53 also interacts with RPKAA1 (AMPK). In addition, PPI network analysis showed that MUC1 interacted with EGFR (HER2-receptor), CTNNB1 (β-catenin), Src, and ICAM-1 (intracellular adhesion molecule)-1.

GO annotation analysis revealed the genetic signal was enriched in 9 MF and 24 BP terms (Supplementary Figure S5). The most significant MF and BP terms were glycoprotein-fucosylgalactoside alpha-N-acetylgalactosaminyl-transferase activity and positive regulation of peptidyl-lysine acetylation, respectively. In addition, glycosylation and AMPK-associated functions or processes were identified. Of the 12 eQTL genes, *MUC1* and *PRKAA1* were the ones that were most annotated to the enriched GO terms.

Gene-drug interactions were identified only for MUC1 and PRKAA1. PRKAA1 interacts with phenformin, metformin, hesperadin, sirolimus, streptozocin, thyroxine, pentostatin, saponarin, fluorinated N,N′-diarylureas, acetylsalicylic acid, and adenosine monophosphate. MUC1 interacted with Huhmfg1 and GO-203-2C (Figure 2).

Based on the CTD database, 66 gene-chemical interactions with 44 chemicals and ten genes were identified (Figure 2; Supplementary Figure S6). Of the ten genes, PRKAA1 and EFNA1 interacted with the largest number of chemicals (17). Oestradiol was six times reported to decrease the expression of EFNA1. AICA ribonucleotide and metformin were five times reported to increase the phosphorylation of PRKAA1. Furthermore, the expression of MUC1 was reported to be increased by the action of aflatoxin B1, oxygen, and valproic acid a total of four times.

## 4 Discussion

In this review, we described the most reported genetic loci that are associated with the increased risk of GC from the available GWAS and conducted meta-analyses and GBA of the genetic variants with available genotypes. Comprehensive meta-analysis and GBA of genetic variants identified 25 significant genes for GC susceptibility. Among the 25 genes, 12 genes (*THBS3, GBAP1, KRTCAP2, TRIM46, HCN3, MUC1, DAP3, EFNA1, MTX1, PRKAA1, PSCA*, and *ABO*) were significant at the gene expression level according to eQTL analysis. To understand the function of these 12 genes, disease network analysis, biological pathway and GO enrichment analysis, and gene-drug and chemical interaction analyses were conducted.

*PSCA* encodes a glycosylphosphatidylinositol-anchored cell membrane glycoprotein. In addition to being highly expressed in the prostate, it is also expressed in the bladder, placenta, colon, kidney, and stomach. *PSCA* is the genetic locus most significantly associated with the risk of *H. pylori*-induced GC in the Japanese population, which is the case with regard to the European population as well (Rizzato et al., 2013). Moreover, in *H. pylori*-infected gastric mucosal tissue, PSCA expression was found to be remarkably suppressed compared to that in normal, non- *H. pylori* infected gastric mucosal tissue (Toyoshima et al., 2018). This can lead to a reduced risk of GC or an increased risk of duodenal ulcers (Tanikawa et al., 2012).

**FIGURE 3:**
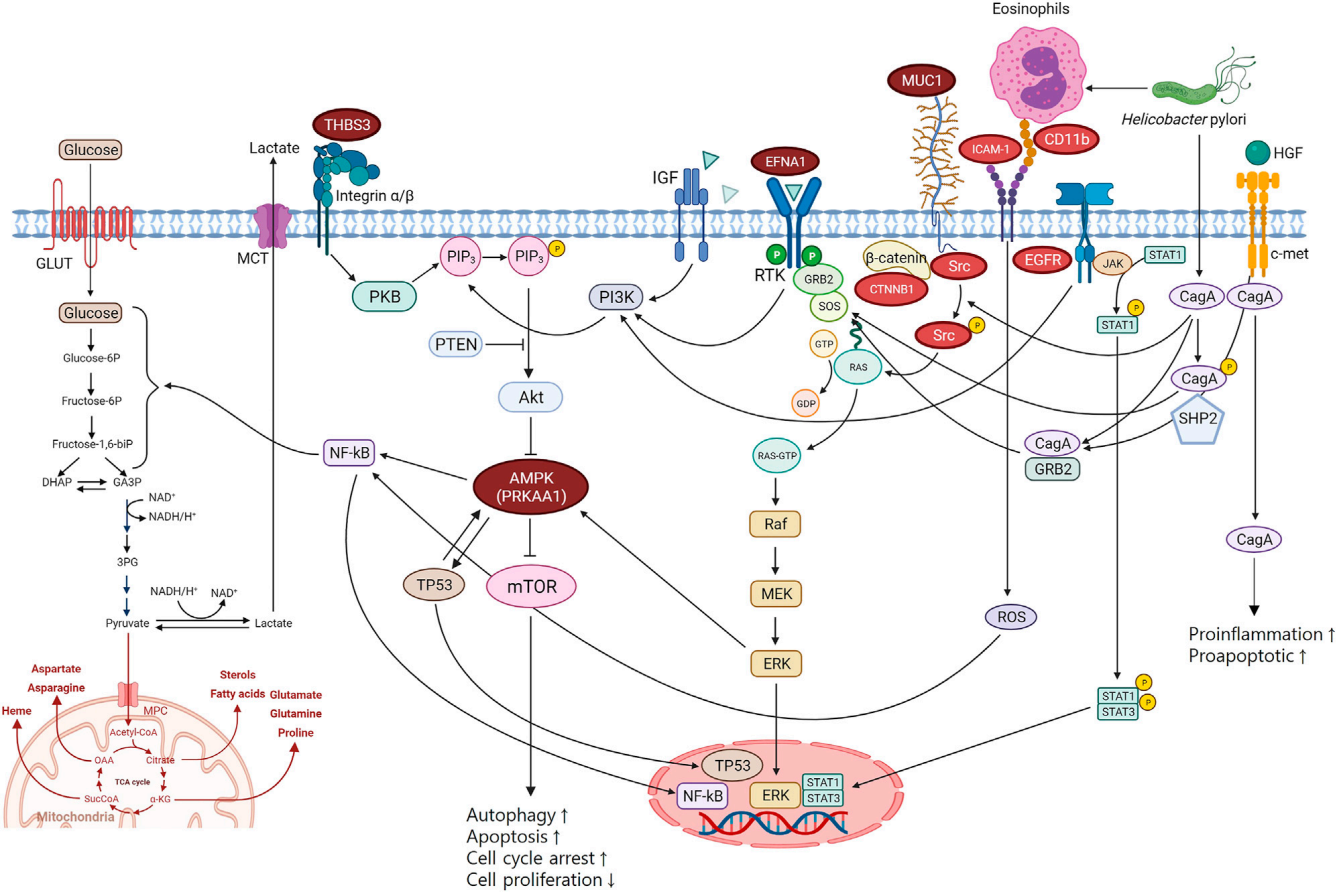
Biological pathways of gastric cancer mechanisms. THBS3, EFNA1, and PRKAA1 are involved in PI3K-Alt-mTOR-signaling pathway which is the key pathway associated with gastric cancer. MUC1 interacted with ICAM-1, CD11b, EGFR, Src, and CTNNB1 in PPI network is a regulator of the PI3K-Alt-mTOR-signaling pathway. PPI, protein-protein interaction.

PRKAA1 belongs to the serine/threonine-protein kinase family. It is the catalytic subunit of AMPK, a cellular energy sensor conserved in all eukaryotic cells (Krishan et al., 2014). PRKAA1 is mainly involved in the PI3K-Alt-mTOR-signaling pathway via AMPK. The PI3K-Alt-mTOR-signaling pathway is a transduction hub linked to various biological pathways and mechanisms associated with carcinogenesis (Figure 3). AMPK negatively modulates mTOR, which plays an important role in regulating cellular energy homeostasis by regulating cellular processes such as protein synthesis and autophagy. mTOR signalling positively regulates cell proliferation and tumorigenesis in various cancers and is often aberrantly activated in cancer. In addition, the PI3K-Alt-mTOR-signaling pathway can influence glycosylation through nuclear factor kappa B (NF-κB), a protein complex that functions as a signal-induced transcription factor regulating proliferation and apoptosis (Magaway et al., 2019; Cho et al., 2021).

The role of PRKAA1 was also confirmed by the GO annotation analysis results (Supplementary Figure S5). PRKKA1 was shown to be associated with affecting the molecular function of AMPK and involved in biological pathways, including glycolysis, PI3K-Akt-mTOR signaling, autophagy, cell cycle, and cell differentiation (Supplementary Figure S5). This confirms that PRKAA1 directly affects the molecular function of AMPK and has a role in its related pathways (Figure 3). In addition, PRKAA1 is also associated with the glucosylceramide process and mitochondrial regulation in biological processes. MUC1, a mucin linked to AMPK’s pathway, seems to be more involved in biological processes through the regulation of protein acetylation, cell adhesion by integrin, and glycosylation (Figure 3; Supplementary Figure S5).

Regarding gene-chemical interactions, 5-aminoimidazole-4-carboxamide (AICA) ribonucleotide is one of the most reported chemicals to phosphorylate PRKAA1 (Supplementary Figure S6) and is widely used as a pharmacological modulator of AMPK activity (Višnjić et al., 2021). In a previous experimental study, AICA ribonucleotide was also shown to induce apoptosis alone in GC cells with the aim of developing a chemotherapy sensitizer for GC (Wu et al., 2016).

Metformin,both as a chemical and drug, had the highest association with PRKAA1 (Figure 2; Supplementary Figure S5), and gene-chemical interaction results revealed that metformin phosphorylates PRKAA1. Metformin is one of the most widely used anti-hyperglycemic drugs for the management of type 2 diabetes. Experimental studies strongly suggest that metformin also possesses anticancer activity mediated through the modulation of several cellular signaling pathways, including AMPK activation and other mechanisms. In addition, metformin use has been associated with a reduced GC risk, where an increasing metformin dose was correlated with a lower GC risk (Kim et al., 2014; Cheung et al., 2019). Regarding gene-drug interactions, phenformin scored four, coming after metformin, which scored five (the score reflects the number of reports in previous studies). It has been reported that both phenformin and metformin can inhibit cell growth through inhibition of cell proliferation, promotion of apoptosis, and cell cycle disturbances (Wang et al., 2018).

EFNA1 is a growth factor that induces cell proliferation, differentiation, and survival by binding to receptor tyrosine kinase (RTK) in the cell membrane to generate Ras-GTP, which activates the mitogen-activated protein kinase (MAPK) pathway in the cytoplasm (Haglund et al., 2007). When extracellular signal-regulated kinase (ERK), an important factor in the MAPK pathway, is activated, the transcription of several genes is activated, thereby resulting in cell growth. RAS mutations lead to sustained activation of the ERK pathway, which leads to cancer development (Mitra et al., 1993; Seger and Krebs, 1995). In addition, ERK is linked to the PI3K-Alt-mTOR-signaling pathway by activating AMPK (Figure 3).

Thrombospondin 3 (THBS3) is an extracellular glycoprotein that mediates cell-to-matrix and cell-to-cell interactions (Mosher and Adams, 2012). THBS3 activates the PI3K-Alt-mTOR-signaling pathway via protein kinase B (PKB).

MUC1 is a single-pass type I transmembrane protein with a heavily glycosylated extracellular domain (Hattrup and Gendler, 2008; Nath and Mukherjee, 2014). MUC1 has been reported to act as an anti-inflammatory molecule in gastric mucosal cells. The anti-inflammatory properties of MUC1 have also been observed in gastric mucosal cell responses to *H. pylori* infection (Guang et al., 2010). In addition, MUC1 inhibits cell proliferation and regulates the PI3K-Alt-mTOR-signaling pathway through a β-catenin-dependent mechanism (Lillehoj et al., 2007).

*H. pylori* induces ICAM-1 and CD11b (integrin) expression, causing degranulation and eosinophil cationic protein (ECP) release (Chmiela et al., 2018). Activation of ICAM-1 by CD11b results in the release of reactive oxidative species, which stimulate NK-κB. In addition, an interaction between the *H. pylori* virulence factor CagA and the receptor c-Met has been found (Eom et al., 2016). CagA stimulates the MAPK/ERK pathway and PI3K-Alt-mTOR-signaling pathway by activating RAS by binding to the c-Met receptor (Churin et al., 2003; Suzuki et al., 2009). Moreover, binding between c-Met and hepatocyte growth factor (HUFF) also stimulates the MAPK/ERK, PI3K-Alt-mTOR-signaling, and JAK/STAT pathways (Jang et al., 2020). These mechanisms converge in inducing cell proliferation, pro-inflammatory response, and cell motility, which are involved in tumor development and progression (Figure 3) (Churin et al., 2003; Suzuki et al., 2009; Bradley et al., 2017).

Our study has some limitations. First, given that only the results of GWAS that have been published were selected, we cannot avoid the possibility of publication bias. In general, GWAS with significant associations are more likely to be published than studies with null associations (Stadler et al., 2010). Second, the SNPs generally reported in GWAS are the lead SNPs with the most significant *p*-value based on Bonferroni multiple tests after LD clumping. However, since lead SNPs are not always causal SNPs, fine-mapping analysis is necessary to investigate the region around the lead SNP to find the presence of other potential causal SNPs (Farh et al., 2015). The GWAS included in our study did not perform such follow-up analyses. Third, the heterogeneity between each GWAS, in terms of population origin, phenotype definition, genotyping platform, and software used can lead to biased results. Since the original data used in the individual GWAS were not available, taking into account sources of variability in the analysis was difficult. Furthermore, the results of GWAS for GC in Caucasians were not included in the GBA due to an insufficient number of SNPs or genes. In the future, the genetic burden of GC in Caucasians and the differences among ethnicities need to be further explored. Lastly, although the meta-analysis results based on the fixed- and random-effect models presented similar estimates for most SNPs, some of the SNPs that were reported only twice in previous studies yielded different estimates. Similarly, SNPs with different estimates between fixed- and random-effect model had high heterogeneity. Given that the random-effect model is more evenly weighted compared to the fixed-effect model (Hedges and Vevea, 1998), it is possible that the estimates of less-reported SNPs are more unstable. In addition, the *p*-value threshold of GWAS is generally 5 × 10^–8^ in discovery and less than 0.05 in validation (Risch and Merikangas, 1996; Oetting et al., 2017). Even if the SNPs were validated multiple times in a single study, the heterogeneity can be highly evaluated because the threshold of *p*-value in validation analysis is high. Thus, some SNPs still require a larger number of external validations to estimate the stable effect size of SNPs associated with GC.

Despite these limitations, our study had several strengths. First, we used a comprehensive and systematic approach to identify all possible GWAS in the literature. Second, the statistical power of our analysis was increased due to the large number of SNPs comprised in the meta-analysis. Moreover, compared to SNP-based GWAS, GBA is more robust in terms of statistical significance. By combining the SNPs of individual GWAS into a gene-based score without increasing the sample size or collecting new data, the statistical power is increased, resulting in a less stringent significance threshold (Liu et al., 2010). Therefore, our study highlights the possibilities that meta-analysis and GBA offer by reusing published summary statistics. Furthermore, functional annotation using disease network, biological pathway, GO, gene-drug, and chemical interaction analyses permitted a further understanding of the mechanisms of GC development.

Based on the comprehensive investigation and multifaceted functional analysis of the reported GC-associated genetic variants, we conclude that *PRKAA*1 is a key gene for GC development. Based on our results, *PRKAA1,* which is involved in the PI3K-Alt-mTOR-signaling pathway, could be a target gene for drug development associated with GC in the future.

## Data Availability

The original contributions presented in the study are included in the article/Supplementary Material, further inquiries can be directed to the corresponding author.
