# Supplementary material for: Systematic review of gastric cancer-associated genetic variants, gene-based meta-analysis, and gene-level functional analysis to identify candidate genes for drug development
Source: Front Genet. 2022 Aug 16;13:928783. doi: 10.3389/fgene.2022.928783 (PMC9446437; doi:10.3389/fgene.2022.928783)
Supplement: Supplementary file 2 [file Presentation1.PPTX]

## Slide 1
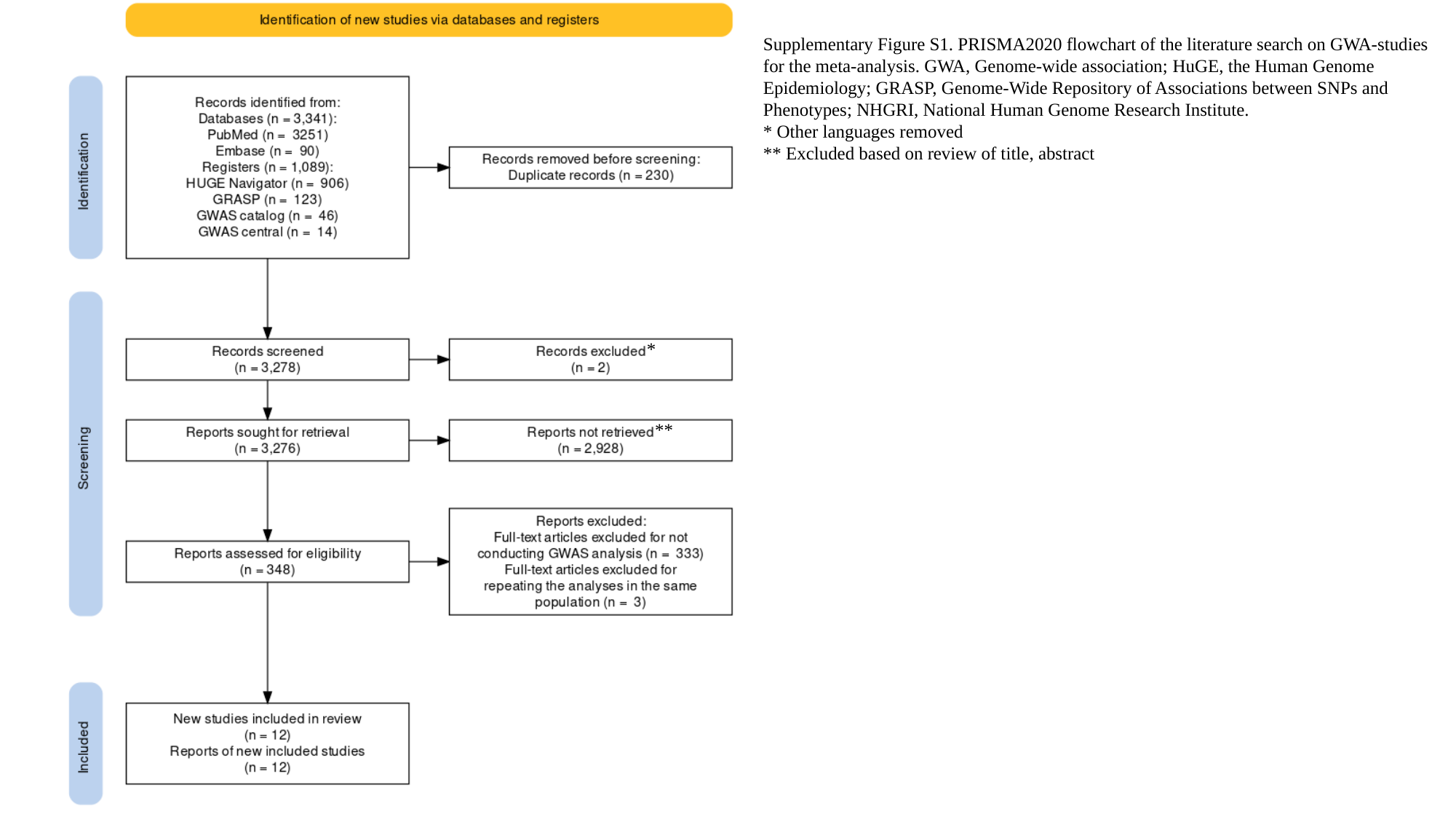

Supplementary Figure S1. PRISMA2020 flowchart of the literature search on GWA-studies for the meta-analysis. GWA, Genome-wide association; HuGE, the Human Genome Epidemiology; GRASP, Genome-Wide Repository of Associations between SNPs and Phenotypes; NHGRI, National Human Genome Research Institute.
* Other languages removed
** Excluded based on review of title, abstract
*
**

## Slide 2
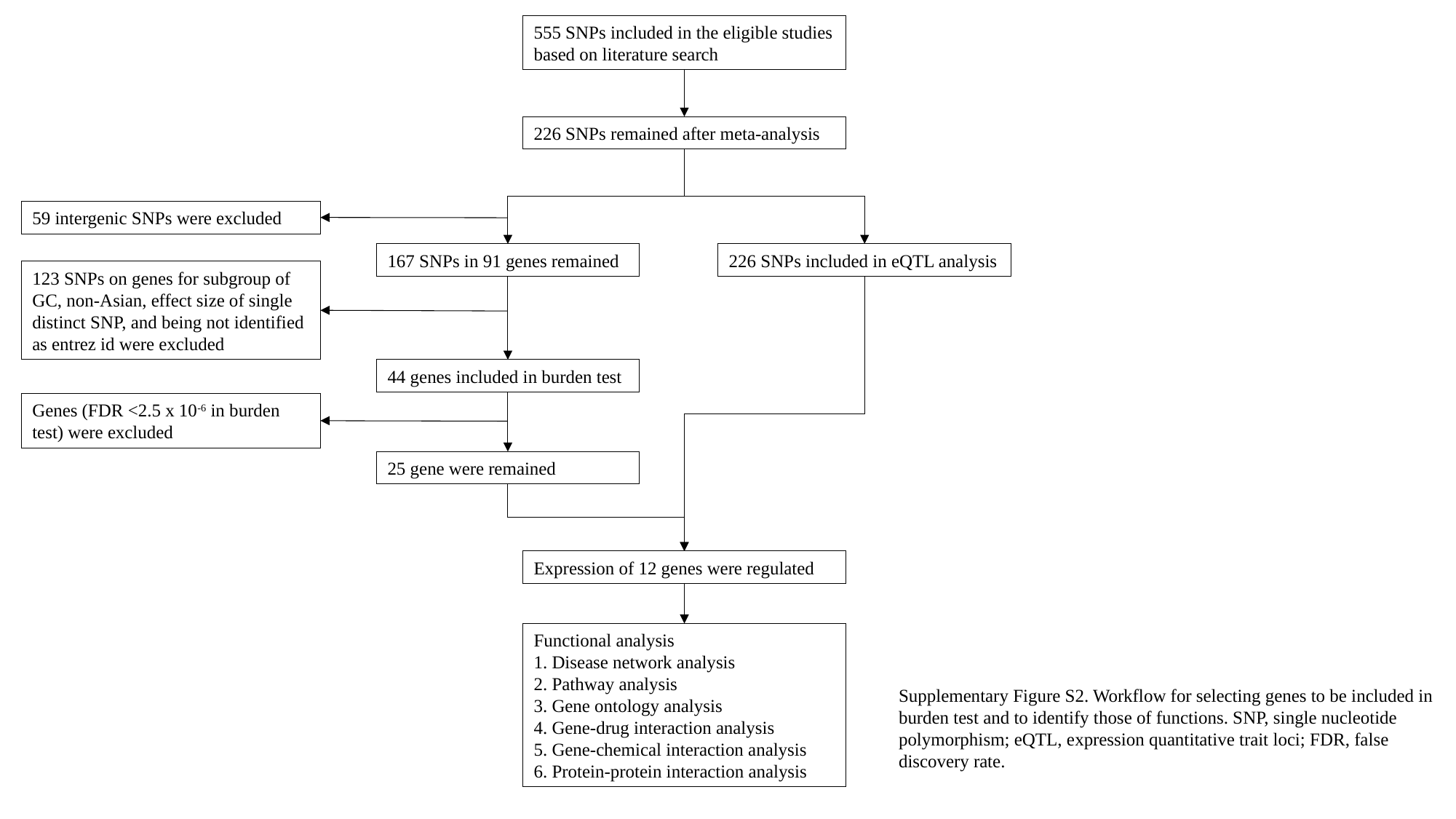

555 SNPs included in the eligible studies based on literature search
226 SNPs remained after meta-analysis
59 intergenic SNPs were excluded
167 SNPs in 91 genes remained
226 SNPs included in eQTL analysis
123 SNPs on genes for subgroup of GC, non-Asian, effect size of single distinct SNP, and being not identified as entrez id were excluded
44 genes included in burden test
Genes (FDR <2.5 x 10^-6^ in burden test) were excluded
25 gene were remained
Expression of 12 genes were regulated
Functional analysis
1. Disease network analysis
2. Pathway analysis
3. Gene ontology analysis
4. Gene-drug interaction analysis
5. Gene-chemical interaction analysis
6. Protein-protein interaction analysis
Supplementary Figure S2. Workflow for selecting genes to be included in burden test and to identify those of functions. SNP, single nucleotide polymorphism; eQTL, expression quantitative trait loci; FDR, false discovery rate.

## Slide 3
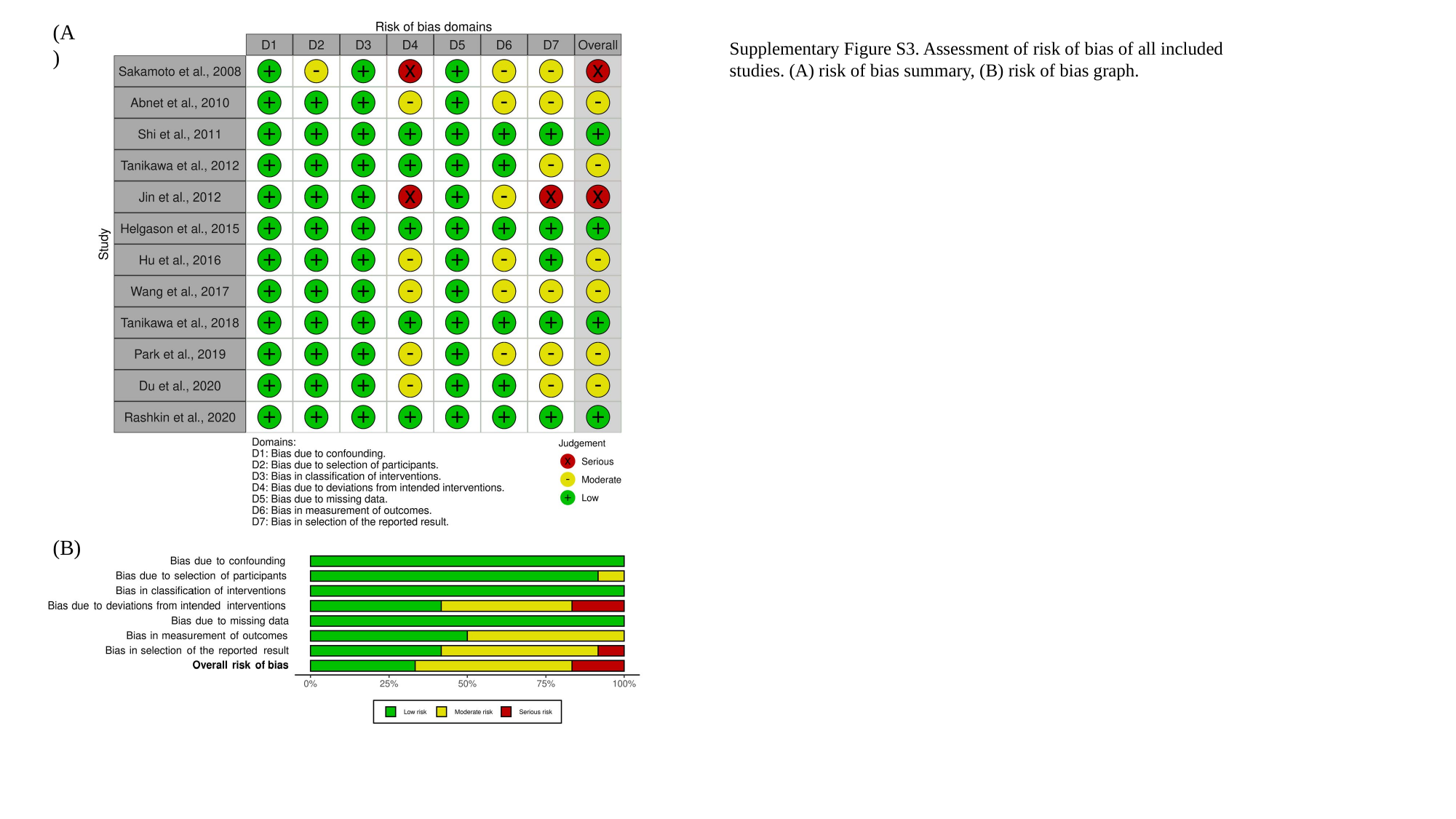

(A)
Supplementary Figure S3. Assessment of risk of bias of all included studies. (A) risk of bias summary, (B) risk of bias graph.
(B)

## Slide 4
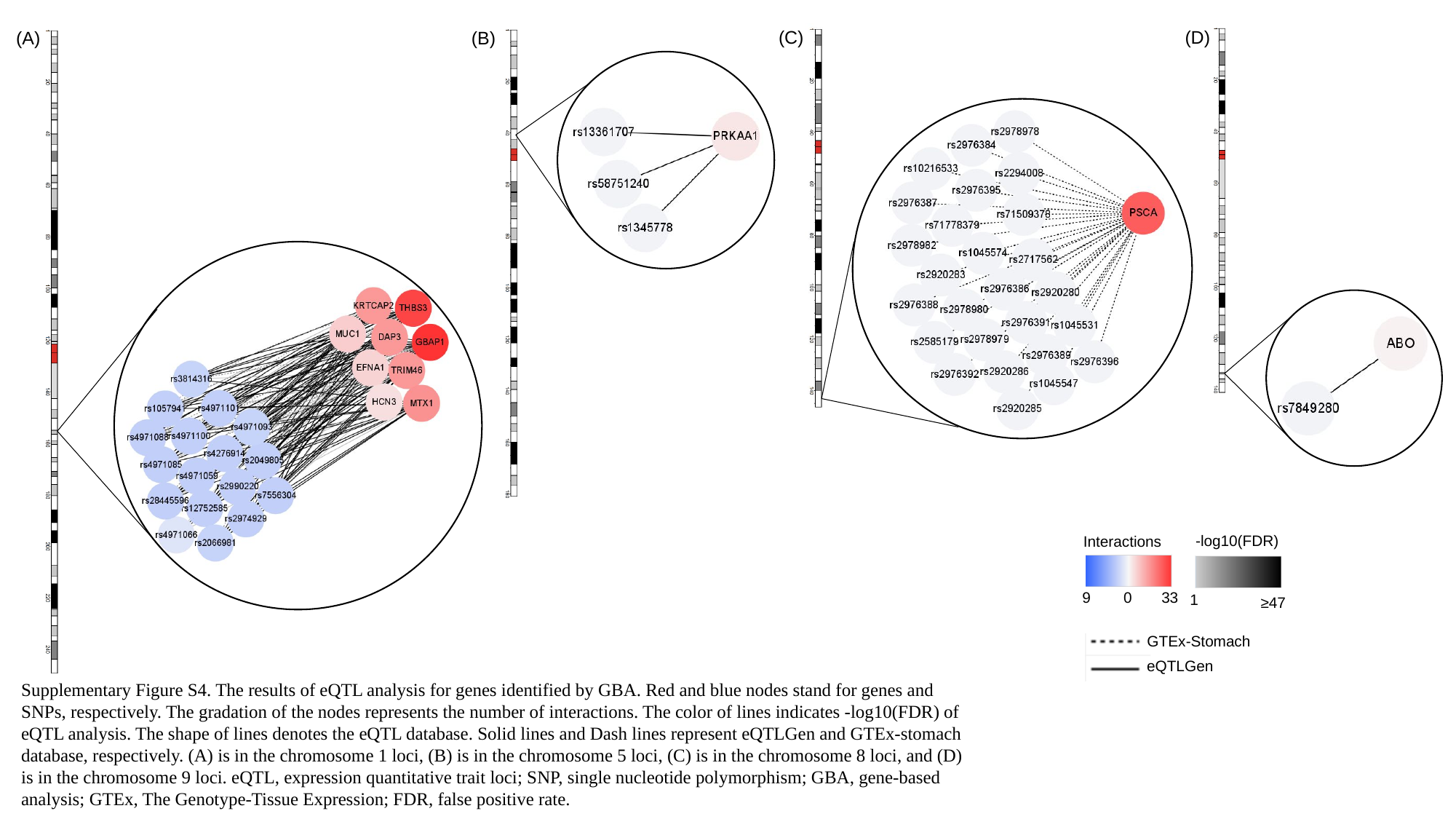

(C)
(D)
(A)
(B)
-log10(FDR)
Interactions
0
9
33
1
≥47
GTEx-Stomach
eQTLGen
Supplementary Figure S4. The results of eQTL analysis for genes identified by GBA. Red and blue nodes stand for genes and SNPs, respectively. The gradation of the nodes represents the number of interactions. The color of lines indicates -log10(FDR) of eQTL analysis. The shape of lines denotes the eQTL database. Solid lines and Dash lines represent eQTLGen and GTEx-stomach database, respectively. (A) is in the chromosome 1 loci, (B) is in the chromosome 5 loci, (C) is in the chromosome 8 loci, and (D) is in the chromosome 9 loci. eQTL, expression quantitative trait loci; SNP, single nucleotide polymorphism; GBA, gene-based analysis; GTEx, The Genotype-Tissue Expression; FDR, false positive rate.

## Slide 5
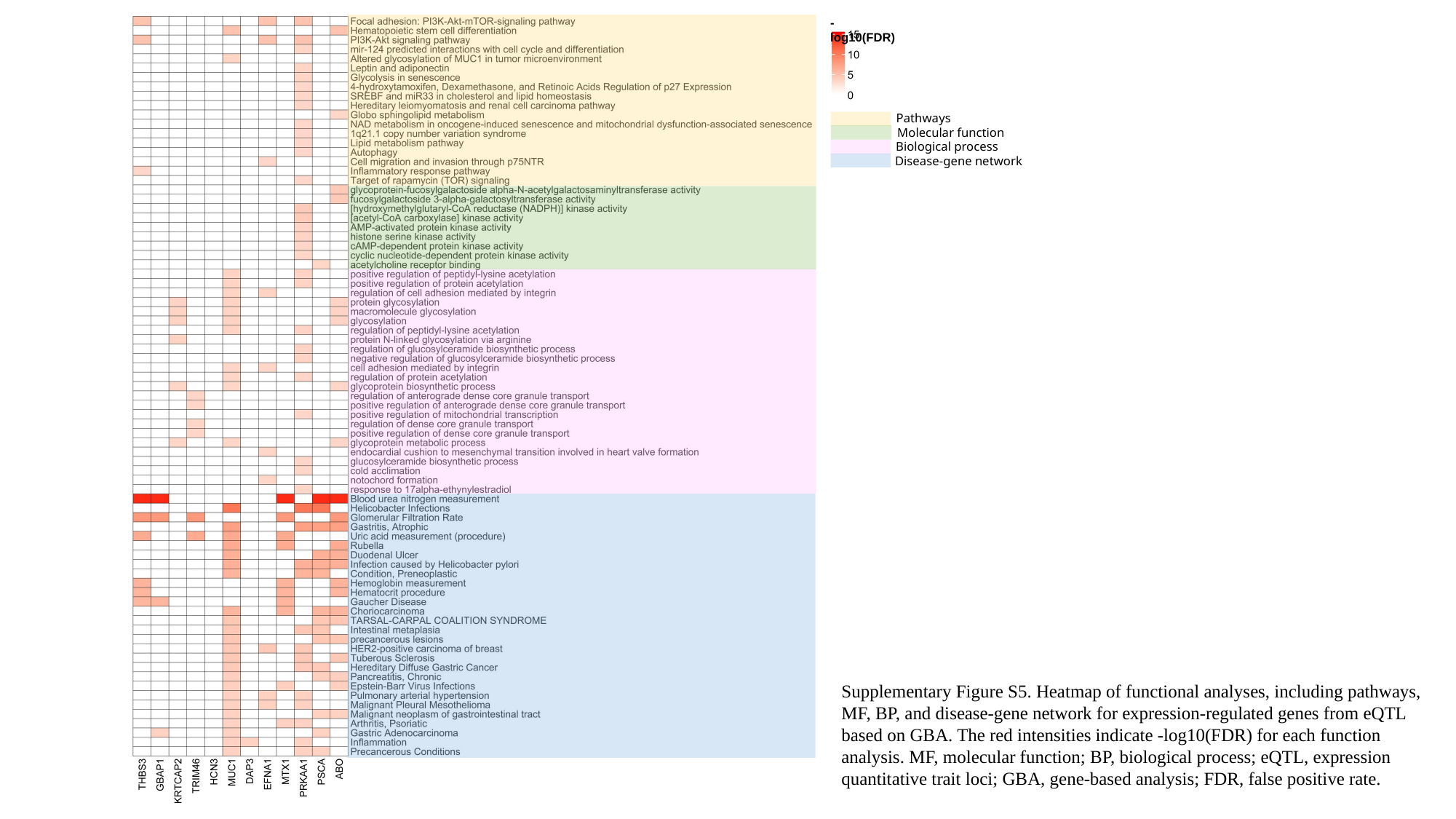

-log10(FDR)
Pathways
Molecular function
Biological process
Disease-gene network
Supplementary Figure S5. Heatmap of functional analyses, including pathways, MF, BP, and disease-gene network for expression-regulated genes from eQTL based on GBA. The red intensities indicate -log10(FDR) for each function analysis. MF, molecular function; BP, biological process; eQTL, expression quantitative trait loci; GBA, gene-based analysis; FDR, false positive rate.

## Slide 6
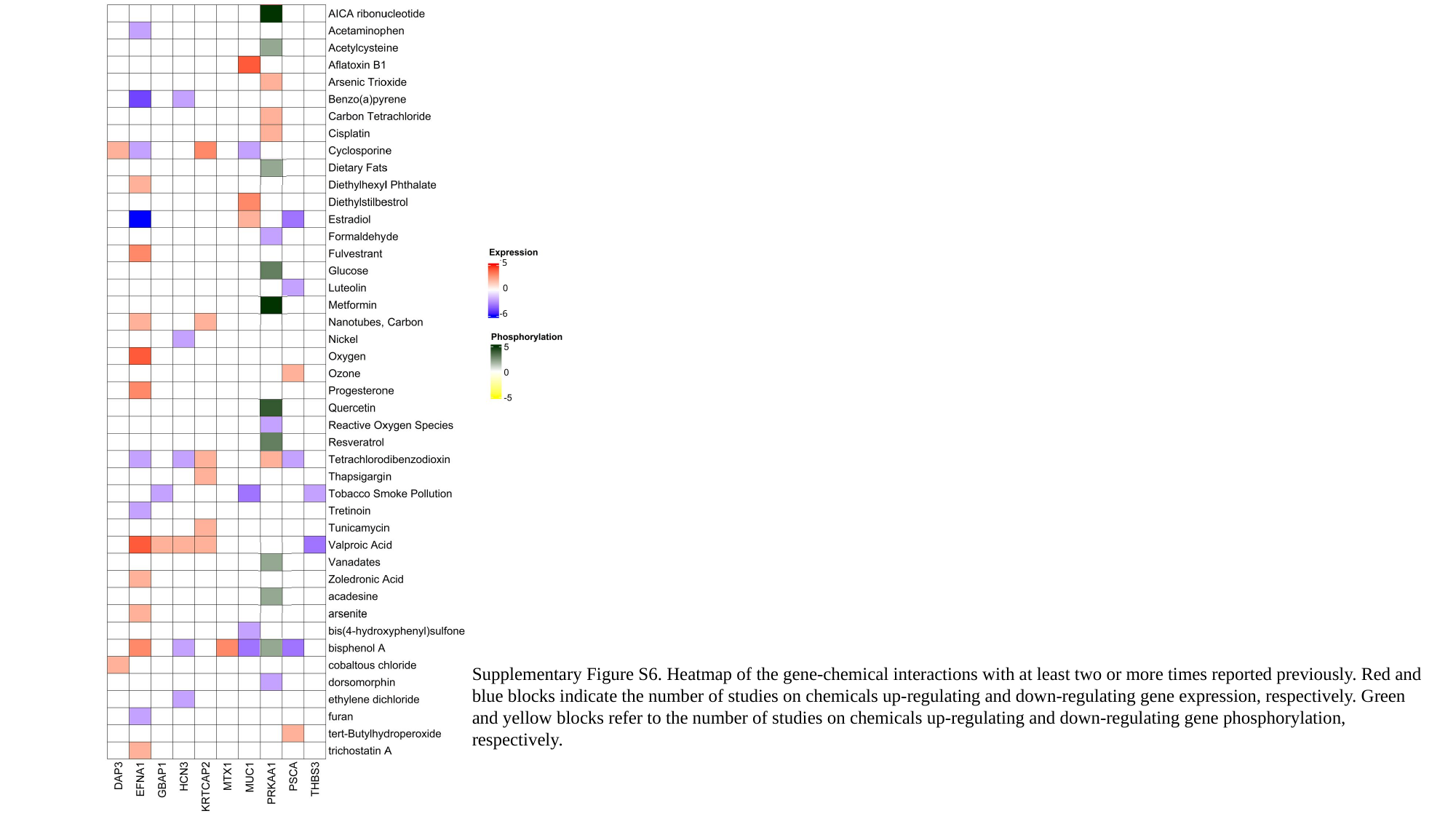

5
0
-6
Supplementary Figure S6. Heatmap of the gene-chemical interactions with at least two or more times reported previously. Red and blue blocks indicate the number of studies on chemicals up-regulating and down-regulating gene expression, respectively. Green and yellow blocks refer to the number of studies on chemicals up-regulating and down-regulating gene phosphorylation, respectively.
