# Supplementary material for: Systematic review of gastric cancer-associated genetic variants, gene-based meta-analysis, and gene-level functional analysis to identify candidate genes for drug development
Source: Front Genet. 2022 Aug 16;13:928783. doi: 10.3389/fgene.2022.928783 (PMC9446437; doi:10.3389/fgene.2022.928783)
Supplement: Supplementary file 4 [file Table2.DOCX]

Supplementary Table S2. Mesh terms for search query and results, literature for review

| **Mesh terms for search query in PubMed** | **Results** |
| --- | --- |
| ((("stomach"[MeSH Terms] OR "stomach"[All Fields] OR "gastric"[All Fields]) OR ("stomach"[MeSH Terms] OR "stomach"[All Fields])) AND ("neoplasms"[MeSH Terms] OR "neoplasms"[All Fields] OR "cancer"[All Fields])) AND (("genome-wide association study"[MeSH Terms] OR ("genome-wide"[All Fields] AND "association"[All Fields] AND "study"[All Fields]) OR "genome-wide association study"[All Fields] OR ("genome"[All Fields] AND "wide"[All Fields] AND "association"[All Fields] AND "study"[All Fields]) OR "genome wide association study"[All Fields]) OR ("genome-wide association study"[MeSH Terms] OR ("genome-wide"[All Fields] AND "association"[All Fields] AND "study"[All Fields]) OR "genome-wide association study"[All Fields] OR "gwas"[All Fields]) OR ("genome-wide association study"[MeSH Terms] OR ("genome-wide"[All Fields] AND "association"[All Fields] AND "study"[All Fields]) OR "genome-wide association study"[All Fields] OR ("gwa"[All Fields] AND "study"[All Fields]) OR "gwa study"[All Fields]) OR (("genome"[MeSH Terms] OR "genome"[All Fields]) AND study[All Fields]) OR (whole[All Fields] AND ("genome"[MeSH Terms] OR "genome"[All Fields]) AND assoication[All Fields] AND study[All Fields]) OR ("genome-wide association study"[MeSH Terms] OR ("genome-wide"[All Fields] AND "association"[All Fields] AND "study"[All Fields]) OR "genome-wide association study"[All Fields] OR ("wga"[All Fields] AND "study"[All Fields]) OR "wga study"[All Fields]) OR ("genome-wide association study"[MeSH Terms] OR ("genome-wide"[All Fields] AND "association"[All Fields] AND "study"[All Fields]) OR "genome-wide association study"[All Fields] OR "wgas"[All Fields])) AND "humans"[MeSH Terms] AND 2000[EDAT] : 2020[EDAT] | PubMed: N=3,251 |
|  |  |
| **Mesh terms for search query in Embase** |  |
| ('single nucleotide polymorphism'/exp OR 'single nucleotide polymorphism') AND ('stomach cancer'/exp OR 'stomach cancer') AND ('genome-wide association study'/exp OR 'genome-wide association study') AND [2000-2020]/py AND [humans]/lim | Embase: N=90 |
