# Supplementary material for: Systematic review of gastric cancer-associated genetic variants, gene-based meta-analysis, and gene-level functional analysis to identify candidate genes for drug development
Source: Front Genet. 2022 Aug 16;13:928783. doi: 10.3389/fgene.2022.928783 (PMC9446437; doi:10.3389/fgene.2022.928783)
Supplement: Supplementary file 5 [file Table5.DOCX]

Supplementary Table S5. A total number of SNPs included in the eligible studies and the meta-analysis.

| **Subtypes** | **SNPs included in the eligible studies based on literature search** | **Duplicated SNPs** | **SNPs remained after meta-analysis** |
| --- | --- | --- | --- |
| Total | 522 | 296 SNPs were previously reported multiple times in different studies or were reported simultaneously in gastric cancer and its subtypes. | 226 |
| Gastric cancer | 323 | 157 duplicated SNPs were reported with multiple previous studies. | 166 |
| Gastric cardia cancer | 36 | 35 SNPs overlapped with SNPs for gastric cancer. | 1 |
| Gastric non-cardia cancer/adenocarcinoma | 98 | 42 SNPs overlapped with SNPs for gastric cancer. | 56 |
| Diffuse-type gastric cancer | 24 | 23 SNPs overlapped with SNPs for gastric cancer. | 1 |
| Intestinal-type gastric cancer | 12 | 12 SNPs overlapped with SNPs for gastric cancer. | 0 |
| Early-onset gastric cancer | 6 | 6 SNPs overlapped with SNPs for gastric cancer. | 0 |
| Late-onset gastric cancer | 6 | 6 SNPs overlapped with SNPs for gastric cancer. | 0 |
| Gastric adenocarcinoma cancer | 17 | 15 SNPs overlapped with SNPs for gastric cancer. | 2 |

SNP, single nucleotide polymorphisms
