# Supplementary material for: Systematic review of gastric cancer-associated genetic variants, gene-based meta-analysis, and gene-level functional analysis to identify candidate genes for drug development
Source: Front Genet. 2022 Aug 16;13:928783. doi: 10.3389/fgene.2022.928783 (PMC9446437; doi:10.3389/fgene.2022.928783)
Supplement: Supplementary file 7 [file Table3.DOCX]

Supplementary Table S3. Risk of bias assessment for non-randomized studies using the Cochrane ROBINS-I tool

| Study | Domain |  |  |  |  |  |  |  |
| --- | --- | --- | --- | --- | --- | --- | --- | --- |
|  | Confounding | Selection of participants | Classification of intervention | Deviation from intervention | Missing data | Measurement of outcomes | Selection of reported results | Overall |
| Sakamoto et al., 2008 | Low | Moderate | Low | Serious | Low | Moderate | Moderate | Serious |
| Abnet et al., 2010 | Low | Low | Low | Moderate | Low | Moderate | Moderate | Moderate |
| Shi et al., 2011 | Low | Low | Low | Low | Low | Low | Low | Low |
| Tanikawa et al., 2012 | Low | Low | Low | Low | Low | Low | Moderate | Moderate |
| Jin et al., 2012 | Low | Low | Low | Serious | Low | Moderate | Serious | Serious |
| Helgason et al., 2015 | Low | Low | Low | Low | Low | Low | Low | Low |
| Hu et al., 2016 | Low | Low | Low | Moderate | Low | Moderate | Low | Moderate |
| Wang et al., 2017 | Low | Low | Low | Moderate | Low | Moderate | Moderate | Moderate |
| Tanikawa et al., 2018 | Low | Low | Low | Low | Low | Low | Low | Low |
| Park et al., 2019 | Low | Low | Low | Moderate | Low | Moderate | Moderate | Moderate |
| Du et al., 2020 | Low | Low | Low | Moderate | Low | Low | Moderate | Moderate |
| Rashkin et al., 2020 | Low | Low | Low | Low | Low | Low | Low | Low |
